# HIV case reporting and HIV treatment outcomes in Qatar

**DOI:** 10.3389/fpubh.2023.1234585

**Published:** 2023-11-03

**Authors:** Elmoubashar Farag, Ivana Bozicevic, Angham Ibrahim Tartour, Hiba Nasreldin, Joanne Daghfal, Sayed Himatt, Mohamed A. Sallam, Ahmed Ismail, Maha Al Shamali, Peter V. Coyle, Alaaeldin Abdelmajid, Naema Al Mawlawi, Mohammed Hamad Al Thani, Hamad Eid Al-Romaihi, Hussam Abdel Rahman Al Soub, Muna Al Maslamani, Abdullatif Al Khal

**Affiliations:** ^1^Public Health Department, Ministry of Public Health, Doha, Qatar; ^2^World Health Organization Collaborating Centre for HIV Strategic Information, University of Zagreb School of Medicine, Zagreb, Croatia; ^3^Communicable Disease Center, Hamad Medical Corporation, Doha, Qatar; ^4^Virology, Hamad Medical Corporation, Doha, Qatar

**Keywords:** HIV, Qatar, case reporting, HIV treatment, viral load

## Abstract

**Aim:**

The aim of the paper is to provide an overview of available HIV case reporting and treatment data for in Qatar for the period 2015–2020.

**Methods:**

HIV case reporting data were analyzed by sex and mode of transmission. To construct HIV care continuum from the data available, we obtained information on the total number of HIV diagnosed patients on antiretroviral treatment (ART) between January 1st 2015 and December 31st 2020, number of patients on ART who had an HIV viral load test and the number who were virally suppressed (defined as having the viral load of less than 1,000 copies/mL).

**Results:**

A total of 515 HIV cases were reported to the Ministry of Public Health since beginning of reporting in 1986, and that included Qatari nationals and expatriate residents diagnosed in Qatar. There was an increase in the annual number of newly reported HIV cases from 16 cases in 2015 (of these, 14 were males) to 58 cases in 2020 (of these, 54 were males). The total number of HIV diagnosed people on ART increased from 99 in 2015 to 213 in 2020. During 2020 the overall viral load testing coverage and viral load suppression among those tested for viral load in men were 72.5% and 93.1%, respectively, while in women these values were 60.4% and 84.4%, respectively.

**Conclusion:**

Due to increase in newly reported HIV cases, there is a need to develop an effective HIV strategic information system in Qatar and data-driven and targeted national HIV response.

## Introduction

Qatar is a country located on the northeastern coast of the Arabian Peninsula. Notably, Qatar is an ethnically diverse country, with approximately 90% of its total population comprising expatriates (1). The estimated number of the population in Qatar in 2021 was 2,725,000 people, of whom 2,046,000 were men and 679,000 women (1). In 2021, it was estimated that the number of the Qatari population in the country was 316,100 while the number of non-Qatari was 2,408,900. Additionally, women account for approximately 51% of the native Qatari population, and 22% of non-native Qataris. This gender imbalance is due to a high number of male migrant workers employed by various industries in Qatar (oil and gas industries, construction work, food industry, etc.).

Notably, health care services in Qatar are provided mainly through the Ministry of Public Health (MoPH) and Hamad Medical Corporation (HMC). HMC is the primary public healthcare provider in Qatar. All people diagnosed with HIV are referred for management and follow-up at the Communicable Diseases Centre (CDC) at HMC, which is the sole provider of outpatient services to these patients in Qatar.

HIV surveillance in Qatar consists of HIV case reporting and monitoring of HIV treatment data. Demographic and behavioural data collected as part of the HIV case reporting system include age, gender, nationality, marital status and mode of HIV transmission. Routine HIV testing in Qatar is carried out at a large scale, for both expatriates and locals, as part of medical check-ups during pre-employment health screening, students applying for a university, before marriage and in certain clinical situations (antenatal care, patients diagnosed with sexually transmitted infections, viral hepatitis and tuberculosis). All foreigners coming to work or reside in Qatar for longer than a month are tested for HIV.

Since October 2018, migrant workers coming from certain countries, including India, Nepal, Sri Lanka, Bangladesh, Philippines, Pakistan, and Thailand have been allowed to test for HIV in designated laboratories in their countries of origin. Only 5% of individuals who were checked prior to arrival are randomly chosen to be re-screened for HIV when they arrive in Qatar. The majority of migrant workers are not re-tested for HIV during the period of their employment in Qatar except for those who work in certain professions such as food handlers, massage centres, beauty salons and barbers. This has caused a decline in the number of HIV tests in Qatar, with the Medical Commission, the largest public provider of HIV testing services, performing 562,807 HIV tests in 2019 compared to 705,968 tests in 2015.

A further decline in HIV testing occurred in 2020 due to the COVID-19 epidemic and restrictions related to entry of new foreign workers to Qatar as of March 2020. Furthermore, HIV testing in Qatar is not particularly targeted towards key populations who bear the highest burden of HIV in all settings (2). Also, there has been a lack of sexual behaviour data in the native Qatari population as well as in foreign workers residing in Qatar.

It is important to note that Qatar is part of the World Health Organization Eastern Mediterranean region (WHO EMR) where the estimated number of people living with HIV (PLHIV) was 420,000 in 2020 (2). There were 44% more new HIV infections in WHO EMR in 2020 compared to 2010, which makes it one of the fastest growing HIV epidemics in the world. Key populations, including men who have sex with men (MSM), sex workers (SW), people who inject drugs (PWIDs), transgender people, and prisoners together disproportionately account for 97% of new HIV infections in the region (3). Still, little data has been published on the HIV epidemic in Qatar (4, 5).

The aim of this paper is to describe the available HIV case reporting and treatment data for the period from January 1st 2015 to December 31st 2020 in Qatar.

## Methods

We obtained HIV data from the MoPH case reporting registry; HIV case reporting to MoPH has been mandated since 1985. Data on patients receiving antiretroviral treatment (ART) were obtained from medical records at the CDC of the HMC.

Electronic HIV case reporting and patient monitoring systems are based on the data collection forms recommended by the WHO. Data, including HIV-related information, is securely stored in a password-protected electronic database with regular backups on a secure server. Quality control steps are taken by well-trained HIV nurses and physicians while doing data collection. Validation, data cleaning and processing is done by the facility senior epidemiologist. The facility senior epidemiologist who is the registry keeper retrieved relevant datasets.

Strict measures, including limited access, are in place to protect sensitive information. Extracted data underwent thorough validation checks to ensure accuracy. The data presented in this report were collected as part of the routine processes within MoPH and HMC for the reporting, testing, and evaluations of HIV cases and care continuum in the State of Qatar, whereby no research question was involved. No personal identifiers were collected nor reported for the purpose of this paper.

We analyzed data on the number of HIV cases reported from 2015 to 2020 to the MoPH, disaggregated by sex. We calculated the proportions of newly reported HIV cases that were diagnosed late (defined as having CD4 counts less than 350 cells/mm^3^ at the time of HIV diagnosis) and in the stage of AIDS at the time of HIV diagnosis (defined as having CD4 counts less than 200 cells/mm^3^ at the time of HIV diagnosis). Additionally, to construct an HIV care continuum from the data available, we obtained from the CDC at HMC information on the total number of HIV diagnosed patients on ART in the 2015–2020 period, number of patients on ART who had an HIV viral load (VL) test and the number who were virally suppressed (defined as having viral load of less than 1,000 copies/mL). The denominator for the VL testing indicator is the number of people on ART, while for the VL suppression it is the number of tested for VL.

Chi-square and Fischer exact tests were used to assess the association between nationality (Qatari versus non-Qatari) and the distribution of newly reported HIV cases in the 2015–2020 reporting period. Chi-square and Fischer exact tests were also used to analyze the differences in the percentage testing for VL and achieving VL suppression by sex in the 2015–2020 period. A value of p of less than 0.05 was set to indicate a significant result. Data were entered in Microsoft Excel and analyzed.

## Results

### HIV case reporting data

A total of 515 HIV cases were reported in Qatar since beginning of reporting, and 217 (42.1%) of those were Qataris. Of these 515 HIV cases, 213 (41.4%) were on ART at the end of 2020, 160 (31.1%) had died and 142 (27.5%) left the country.

In 2020, there were 2.0 newly reported HIV cases per 100,000 population in Qatar.

As shown in Table 1, since 2015 there was more than a three-fold increase in the number of reported HIV cases, from 16 cases in 2015 to 58 cases in 2020. Of these, in 2020 there were 21 cases reported in Qatari and 37 in non-Qatari nationals. Additionally, there was a pronounced increase in the male-to-female ratio in newly reported cases among the Qatari population, ranging in the 2015–2020 period from 71 in 2015 to 13.5:1 in 2020. Similarly, in the non-Qatari population male HIV cases substantially outnumber female cases. Meanwhile, the male-to-female ratio in the total population in Qatar in the 2015–2020 period ranged from the highest of 3.1:1 in 2015 and 2016, to the lowest of 2.5:1 in 2020.

**Table 1:** Number of newly reported HIV cases in Qatari and non-Qatari nationals, total number of people diagnosed with HIV in Qatar who were on antiretroviral treatment (ART) and viral load testing outcomes, 2015–2020.

	2015		2016		2017		2018		2019		2020	
	M	F	M	F	M	F	M	F	M	F	M	F
Number of newly reported HIV cases in Qatari nationals	4	2	5	1	7	2	13	1	15	1	20	1
Number of newly reported HIV cases in non-Qatari nationals	10	0	11	1	17	0	21	4	31	7	34	3
Total number of patients on ART	64	35	71	37	82	39	103	44	125	51	160	53
Total number tested for VL (% tested for VL among those on ART)	58 (90.6)	26 (74.3)	53 (74.6)	32 (86.5)	70 (85.4)	32 (82.0)	95 (92.2)	39 (88.6)	107 (85.6)	41 (80.3)	116 (72.5)	32 (60.4)
Total number with VL <1,000 copies/mL (% of those with VL < 1,000 copies/mL among those tested for VL)	42 (72.4)	21 (80.8)	36 (67.9)	21 (65.6)	44 (62.9)	21 (65.6)	75 (78.9)	34 (87.2)	96 (89.7)	36 (87.8)	108 (93.1)	27 (84.4)

M, Male; F, Female; HIV, Human Immunodeficiency Virus; ART, Antiretroviral Treatment; VL, Viral Load.

A statistically significant difference was observed between Qataris and non-Qataris in the distribution of newly reported HIV cases in the 2015–2020 period (*X*^2^ = 530.3, *p* < 0.05), with non-Qataris contributing with the higher proportion in newly reported cases.

Furthermore, in 2020, at the time of diagnosis, 60.3% of HIV cases had a CD4 count of <350 cells/mm^3^, while 29.3% had the CD4 count of <200 cells/mm^3^. Reportedly, a small number of people died from HIV in the 2015–2020 period, ranging from no deaths in 2015 to three in 2017 and 2018, respectively.

The majority of HIV cases in the 2015–2020 period had sexual transmission reported as the mode of infection and were aged 25–49 years. No case was attributed to injecting drug use while there were 1–3 cases annually in the 2016–2019 period whose mode of transmission was recorded as unknown. There was only one case of mother-to-child HIV transmission in 2019. Regarding the sex of cases, the number of male cases increased from 14 in 2015 to 54 in 2020. Notably, in 2020, five out of 20 newly reported male cases in Qatari nationals were in the age group 15–24 years old.

### HIV care and patient monitoring data for 2015–2020

As shown in Table 1, the total number of people on ART increased from 99 persons in 2015 to 213 in 2020. The percentage of patients on ART tested for VL was somewhat above 90% in men in 2015 and 2018, while in females it ranged from the lowest value of 60.4% in 2020 to the highest of 88.6% in 2018. Notably, VL testing coverage was the lowest in 2020.

In the 2015–2018 period, VL suppression in ART patients tested for VL was suboptimal, with the lowest values reported in 2017 (62.9% in males and 65.6% in females). In 2019 and 2020, VL suppression was around 90% in men, while in women it was 87.8 and 84.4%, respectively. There was no statistically significant difference in the proportion of cases tested for VL and achieving VL suppression by sex in the 2015–2020 period (*X*^2^ = 4.5, *p* = 0.95).

## Discussion

Available data indicate a substantial increase in reported HIV cases in Qatar since 2015. In addition, there was a high male-to-female ratio in reported cases, ranging from 7:1 in 2015 to 13.5:1 in 2020 while the male-to-female ratio in the total population in Qatar ranged from the highest of 3.7:1 in 2016 to the lowest of 2.7:1 in 2020. This might indicate an increase in male-to-male HIV transmission, or heterosexual transmission with women not being tested and reported. According to reports by healthcare providers, a number of these infections in men may have also occurred during vacation time spent in the countries of their origin. However, no behavioural data nor phylogenetic analysis are currently available to confirm this.

Furthermore, our data for 2020 reveal a high proportion of people who were diagnosed with HIV late. This could entail potential adverse public health implications related to increased risk of HIV transmission in the community and greater healthcare expenses (6). Notably, the later presentation of HIV in Qatar is multifaceted, stemming from a complex interplay of factors.

For instance, many people may remain unaware of the risk factors contributing to their susceptibility to HIV or may have limited understanding of the virus and its modes of transmission. Additionally, HIV infection can often assume an asymptomatic or inconspicuous presentation, characterized by mild, influenza-like symptoms that may be readily disregarded or misattributed to alternative medical conditions. Consequently, this can cause delays in seeking medical attention and impede timely diagnosis.

Due to lack of HIV and behavioural data in key populations and in migrant workers, it is not possible to understand key sources of HIV infections in Qatar, which also hinders development of a targeted and effective HIV response. Additionally, there are no estimates of the number of PLHIV in Qatar, which hinders the constructions of the full HIV care cascade and understanding how many PLHIV are diagnosed.

Free access to high-quality ART for HIV diagnosed individuals has been one of the most successful components of the National HIV Program in the country but challenges exist. Data indicate sub-optimal VL testing coverage and VL suppression, despite readily available testing and treatment, which is more pronounced in women. This indicates the need to identify barriers to more effective VL testing and adherence to ART. Lower coverage with HIV VL testing in 2020 compared to earlier years is likely to be due to barriers in accessing health care services brought by the COVID-19 epidemic (7). The lower VL testing coverage might result in a failure to detect patients that are not virally suppressed and might subsequently lead to increases in ART resistance (8). However, HIV drug resistance and treatment failure remain very low in Qatar.

ART drugs in Qatar are directly purchased from pharmaceutical companies via HMC, and access to ART is free of charge for all patients regardless of nationality. There is no structured program of pre-exposure HIV prophylaxis (PrEP) for key populations and PrEP is given to a small number of persons attending the clinic for sexually transmitted infections of the HMC and occasionally to contacts of HIV infected patients. Both TDF-FTC (Truvada) and TAF-FTC (Descovy) are used.

The limitations of our analysis includes relatively short period of data collection and the use of only one data source, which is HIV case reporting and patient monitoring. Nevertheless, it provides evidence on the newly reported HIV cases and indicates potential increase in HIV transmission that should be further assessed by enhancing the existing surveillance system, particularly in terms of data availability. Among the limitations is also the inability to show data on the estimated number of PLHIV and the number of HIV diagnosed persons in Qatar. Estimates of the number of PLHIV using Spectrum or other WHO-recommended tools have not yet been done in Qatar. The current HIV surveillance system cannot provide information on the total number of persons diagnosed and living with HIV in Qatar. Therefore, substantial improvements are needed in terms of data availability to better understand these basic outcomes of the continuum of HIV testing and treatment. To understand behavioural characteristics of the HIV epidemic in Qatar and obtain population-level HIV data, it is necessary to conduct HIV bio-behavioural surveys in populations at higher risk (9).

Development of impactful HIV testing services requires adoption and implementation of innovative and efficient ways to offer confidential and readily accessible HIV testing and counseling to those at increased risk, via community-based testing, partner notification services, social network-based testing and HIV self-testing (10). Importantly, public health response to HIV should include measures to combat all forms of stigma and discrimination, which may have been attributed to the decreased rate of testing in the country, with particular attention to the needs of the most vulnerable and hardest to reach.

Qatar committed to contribute US$50 million for global efforts to end the HIV epidemic in the 2020–2022 period during the Global Fund’s Sixth Replenishment conference, which is five-fold increase from its contribution made for the previous replenishment (11). This is an indication of raising awareness of HIV among policy makers as a global and a national health challenge.

## Conclusion

There has been a substantial increase in reported HIV cases in Qatar from 2015 to 2020, and the majority of reported cases were men. This necessitates development of an effective HIV strategic information system to identify factors behind this increase, which will enable the implementation of data-driven and targeted national HIV response.

## Data availability statement

The datasets presented in this article are not readily available due to ethical restrictions. Requests to access the datasets should be directed to Dr. Elmobashar Farag (eabdfarag@moph.gov.qa).

## Author contributions

EF, HN, SH, HS, AK, AT, and IB reviewed the literature. JD and AK acquired the data. EF, AK, AT, and IB drafted the article, and all authors participated in the planning, conception of the manuscript, interpreting the data, and critically revising the manuscript. All authors contributed to the article and approved the submitted version.

## Acknowledgments

This work was supported by the Ministry of Public Health, Qatar, grant MOPH/TAC/PHD/092/2021.
